# Clinical and dosimetric experience with MammoSite‐based brachytherapy under the RTOG 0413 protocol

**DOI:** 10.1120/jacmp.v8i4.2654

**Published:** 2007-10-24

**Authors:** Jadwiga B. Wojcicka, Donette E. Lasher, Ronald Malcom, Gregory Fortier

**Affiliations:** ^1^ Department of Radiation Oncology York Cancer Center York Pennsylvania U.S.A.

**Keywords:** MammoSite, brachytherapy, breast cancer, high dose rate, partial breast irradiation

## Abstract

MammoSite balloon brachytherapy is a relatively new technique for partial breast irradiation. The present paper focuses on the treatment planning, dosimetry, and quality assurance aspects of that treatment, based on the Radiation Therapy Oncology Group 0413 randomized prospective trial (RTOG 0413) protocol. We investigate the usefulness of evaluating implants for treatment appropriateness according to the full set of RTOG criteria as compared with the manufacturer's guidelines. We describe our methods to improve MammoSite balloon implants that would otherwise not comply with the protocol. The initially acquired computed tomography (CT) images are evaluated for tissue conformance, balloon surface–to–skin distance, and balloon symmetry. If the implant fails to meet the foregoing criteria, corrective action such as delay in the CT scan, balloon manipulation, or fluid volume adjustment is taken, and the patient is re‐scanned. If the corrective action appears to be successful, three dimensional treatment planning and dose–volume histogram analysis is performed to evaluate the geometric and dosimetric parameters with regard to the RTOG 0413 protocol. The evaluated parameters include

• volume ratio of the lumpectomy cavity to the ipsilateral breast,

• target volume coverage,

• tissue–balloon conformance,

• balloon symmetry,

• minimal balloon surface–to–skin distance,

• maximum skin dose, and

• normal breast tissue dose–volume parameters *V*150 and *V*200.

Among our implants, 21.7% did not initially meet the RTOG 0413 acceptance criteria. Asymmetry and poor conformance values reduce the target volume coverage, and so an implant with moderate conformance and asymmetry can be within the manufacturer's guidelines, but still not meet the RTOG criteria. Our intervention corrected all but one of the implants that failed to meet the criteria. Manipulating the cavity and adjusting the balloon volume may salvage an implant and meet the strict geometric and dosimetric criteria imposed by the RTOG 0413 protocol.

PACS number: 7.53.Jw

## I. INTRODUCTION

Breast‐conserving therapy (BCT) is an accepted treatment option for early‐stage breast cancer. Clinical data with long‐term follow‐up indicate that BCT provides overall survival that is equivalent to that for modified radical mastectomy.^(^ ^1^ ^,^ ^2^ ^)^

The typical BCT regimen is a course of whole‐breast irradiation, using external‐beam radiotherapy in 5 weekly fractions for 6–7 weeks. Other studies report that BCT is underutilized in certain patient populations.^(^ ^3^ ^–^ ^5^ ^)^ The time commitment required for this treatment regimen has been suggested to be one of the reasons that many patients do not receive BCT.^(6)^ Several promising studies indicate that accelerated partial breast irradiation (PBI) to the tumor bed provides local control that is equivalent to the standard therapy in a carefully selected group of patients.^(7)^ The PBI technique offers the advantage of shortened treatment duration and increased patient convenience.

The National Surgical Adjuvant Breast and Bowel Project (NSABP) and the Radiation Therapy Oncology Group (RTOG) have combined efforts and opened a phase III randomized trial (NSABP B‐39/RTOG 0413)^(7)^ to evaluate the effectiveness of PBI as compared with the broadly accepted technique of whole‐breast irradiation in providing equivalent local tumor control in the breast following lumpectomy for early‐stage breast cancer. The PBI techniques included in the trial are multi‐catheter interstitial brachytherapy, three‐dimensional conformal radiation therapy (3D‐CRT), and intracavitary brachytherapy using the MammoSite balloon (Cytyc Corporation, Palo Alto, CA).

The PBI technique using external‐beam 3D‐CRT is readily available to most clinics. However, localization and immobilization uncertainty require increased treatment margins in 3D‐CRT as compared with those in brachytherapy. In addition, normal tissues receive entrance and exit dose with 3D‐CRT.

Although interstitial brachytherapy PBI has been widely used and has the longest clinical follow‐up, it is a technically more complex procedure that is highly operator‐dependent. The simpler single‐catheter MammoSite design with a balloon that expands to fill the lumpectomy cavity has allowed for broader use of the PBI technique and was approved by the U.S. Food and Drug Administration (FDA) in May 2002. Clinical results from the initial experience and the dosimetry achieved with the MammoSite device have been reported.^(^ ^8^ ^,^ ^9^ ^)^

The RTOG 0413 protocol contains specific geometric and dosimetric criteria for appropriateness of treatment with MammoSite. Evaluation of the implant according to the criteria requires computed tomography (CT)–based 3D treatment planning. The balloon, the planning target volume (PTV), breast tissue, air, and seroma must be contoured for dose–volume histogram (DVH) analysis. The labor involved in this planning varies depending on the treatment planning system in use. The manufacturer also provides similar, but less extensive, criteria for appropriateness of treatment. These criteria can be evaluated with less contouring and without any DVH analysis, potentially saving treatment planning time.

The present paper uses the RTOG 0413 protocol criteria to focus on the treatment planning, dosimetry, and quality assurance aspects of treatment. Treated patients were not a part of the protocol. We investigated the usefulness of evaluating the implants according to the full set of RTOG criteria as compared with the manufacturer's guidelines. We describe our methods to improve MammoSite balloon implants that otherwise would not comply with the protocol.

## II. MATERIALS AND METHODS

During 2006 and 2007, we used the MammoSite technique to treat 22 patients, following our Institutional Review Board protocol. Eligibility criteria were determined using a combination of recommendations from the American Brachytherapy Society (ABS) and the American Society of Breast Surgeons (ASBS).^(^ ^10^ ^,^ ^11^ ^)^ Patients were considered eligible for this procedure if they met all of the following criteria:

- Underwent lumpectomy with negative margins by at least 2 mm
- Showed negative sentinel lymph node sampling if no axillary dissection was done
- Diagnosed as pathologic stage N0 if disease was invasive
- Had a tumor of ≤2cm in diameter
- Showed no lymphovascular invasion
- Showed no multicentric disease

Eligibility requirements also included an age of 45 years or more. The initiation of brachytherapy had to occur within 6 weeks of the final breast surgery. All patients gave informed consent before entry.

### A. Technique

**Figure 1:**
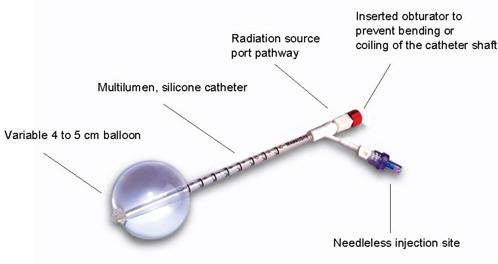
The MammoSite balloon with components identified. (Image provided courtesy of Cytyc Corporation and affiliates.)

The MammoSite device consists of dual‐lumen catheter and a silicone balloon (Fig. 1). The external lumen is the inflation channel and the central lumen accommodates insertion of the high‐dose ${}^{192}\text{Ir}$ source. The spherical balloon is currently available in two sizes: 4 – 5 cm diameter and 5 – 6 cm diameter, with fill volumes ranging from 34 cm^3^ to 70 cm^3^ and from 70 cm^3^ to 125 cm^3^ respectively.

At our institution, the MammoSite catheter is placed during the postoperative ultrasound‐guided implantation procedure. The balloon is inflated with saline solution mixed with small amount of radiographic contrast (up to 10%) to aid in visualization. The inflation size is chosen to completely fill the cavity and ensure conformance of the tissue to the balloon. At 24 – 48 hours post implant, a CT scan with 3‐mm slice thickness and spacing is obtained using a GE Advantage CT scanner (GE Healthcare, Giles, U.K.). The images are initially evaluated for treatment appropriateness according to the manufacturer's recommendations: tissue conformance, balloon surface–to–skin distance, and balloon symmetry.

In an ideal implant, the balloon would be in contact with all surfaces of the lumpectomy cavity. However, air and seroma can occupy the space between the cavity and the balloon, displacing the target volume from the prescription dose. Tissue—ballon conformance is estimated by measuring the air pockets and seroma volumes trapped between the balloon surface and the lumpectomy cavity and expressing those measurements as a percentage of the 1‐cm spherical annulus volume around the balloon. The manufacturer recommends that the ratio be less than 10%. The minimal balloon surface–to–skin distance should be 7 mm; however, the distance may be as low as 5 mm for a maximum of 1 continuous centimeter in the inferior–superior direction. The intent of the foregoing recommendation is to maintain the skin dose below 145% of the prescription dose. Balloon symmetry is defined by the 2‐mm maximum deviation of the central dwell position from the balloon center, which will keep the dose distribution within the American Association of Physicists in Medicine (AAPM) Task Group (TG) 40 recommendation of ±15% ^(12)^ at the prescribed distance from the balloon. The manufacturer's criteria are a subset of the RTOG criteria described in Table 1. The necessary evaluation can be rapidly performed at the CT scanner.

**Table 1:** Radiation Therapy Oncology Group (RTOG) 0413 protocol criteria for the MammoSite implant

RTOG 0413 criteria	Tolerance
(Lumpectomy cavity) / (whole breast reference volume), assuming lumpectomy cavity = balloon	≤30% based on postoperative computed tomography volume
Target volume coverage^a^	≥90%
Tissue–balloon conformance:	
Volume of trapped air and fluid as percentage of PTV_EVAL	<10% of the PTV_EVAL
Balloon symmetry	≤2 mm
Minimum balloon surface–to–skin distance	≥7 mm or maximum skin dose
Maximum skin dose	≤145% of prescription dose
Normal breast tissue dose–volume parameters:	
*V*150	$V150\leq 50\;{\text{cm}}^{3}$
*V*200	$V200\leq 10\;{\text{cm}}^{3}$
Uninvolved normal breast	<60% of the whole breast reference volume should receive≥50% of the prescription dose

a (%PTV_EVAL coverage) – [(volume of trapped air or fluid/volume of PTV_EVAL)×100]=≥90%.

If the implant fails to meet the foregoing criteria, corrective action such as that suggested by Vu et al.^(13)^ is taken, and the patient is re‐scanned. Possible actions include delaying the planning and repeating the CT scan in a few days, massaging the cavity, and adjusting the fluid volume. The delay gives the air and seroma time to resolve without further intervention. A small volume of fluid can be temporarily removed from the balloon before massage of the cavity to remove air and seroma. The fluid volume can also be adjusted in an attempt to improve conformity or symmetry. If the corrective action is successful, and if the initial criteria outlined earlier are fulfilled, the CT images are sent to a Plato (Nucletron, Veenendaal, Netherlands) brachytherapy planning system (BPS).

Initially, the patient is simulated with a dummy source cable inserted into the MammoSite catheter to locate the distal end of the catheter and the first dwell position. Based on the bisector technique described by Edmundson and colleagues,^(9)^ the center of the balloon is geometrically located. The distance between the source first dwell position in the catheter and the balloon geometric center (called the “offset”) is measured. Next, the Source Position Simulator (SPS) dummy wire is inserted through the transfer tube into the MammoSite catheter, and the catheter total treatment length is measured. The treatment length to the center of the balloon is calculated as the total treatment length minus the offset. The SPS dummy wire is sent to this position, and the initial reference film is taken. The balloon diameter is measured and compared with the fill volume reported by the surgeon and is also checked against manufacturer's tables.

### B. RTOG 0413 geometric and dosimetric parameters

**Figure 2:**
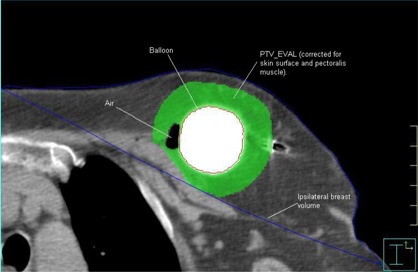
RTOG 0413 volumes of interest. The balloon, PTV_EVAL, ipsilateral breast, and air volumes are depicted. The balloon is contoured at the edge of the contrast medium. PTV_EVAL is a 1‐cm expansion from the balloon surface, excluding the balloon volume, the chest wall and pectoralis muscle, and the first 5 mm inside the skin surface. Air pockets inside the treatment volume are contoured, but air pockets within the balloon are not included. The ipsilateral breast is contoured as are all tissues within the standard tangential field borders, except the lung.

Fig. 2 illustrates the RTOG volumes. The lumpectomy cavity is defined on CT scan as the edge of the contrast media within the balloon. Because the implanted balloon moves with the target, compensation for variability in the treatment set‐up is not necessary, and the clinical target volume (CTV) equals the PTV, which equals the PTV_EVAL. (The PTV_EVAL is defined as the breast tissue volume bounded by the automatic expansion from the edge of the balloon by 1.0 cm in all directions, minus the balloon volume.) Expansion of the PTV_EVAL is limited to breast tissue only (chest wall, pectoralis muscle, and skin surface are not included). In addition, the volume of ipsilateral breast is outlined and the lumpectomy cavity is subtracted from the breast volume per RTOG 0413. Air and fluid (seroma) volumes (AFVs) are outlined, and balloon conformity (a percentage) is calculated as AFV/PTV×100. Balloon symmetry is verified on the axial, coronal, and sagittal views using the multiplanar reconstruction tool in the Plato BPS. Skin points are defined as the closest points on the patient's skin to the balloon's surface.

The dose is prescribed at a distance of 1.0 cm from the balloon surface, in a plane transverse to the MammoSite catheter at the balloon center. The Nucletron BPS is used to perform the dwell‐time calculation. Treatment is delivered by a high dose rate ${}^{192}\text{Ir}$ source in 10 fractions of 340 cGy per fraction, administered twice daily, for a total of 3400 cGy.

Geometric and dosimetric parameters are evaluated according to the RTOG 0413 protocol. The parameters include

- volume ratio of the lumpectomy cavity to the ipsilateral breast,
- tissue–balloon conformance,
- balloon symmetry,
- minimal balloon surface–to–skin distance,
- maximum skin dose, and
- volume of normal breast tissue receiving 150% and 200% of the prescribed dose (*V*150 and *V*200), and target volume coverage (*V*90).

Target volume coverage must be greater than or equal to 90% as calculated by the formula

(1) $$TargetVolumeCoverage=V90_{PTV\_EVAL}-\frac{V_{air}+V_{seroma}}{V_{PTV\_EVAL}},$$

where $V90_{\text{PTV}\_\text{EVAL}}$ is the volume of PTV_EVAL receiving 90% of the prescription dose, and $V_{\text{air}},V_{\text{seroma}}$, and $V_{\text{PTV}\_\text{EVAL}}$ are respectively the volumes of air, seroma, and PTV_EVAL.

Table 1 presents the parameters and their tolerance levels.

### C. Quality assurance

To assure the continued integrity of the balloon throughout treatment, the balloon diameter and treatment dwell position as compared with the balloon center are verified before each fraction is delivered. Before each consecutive fraction, a film with the SPS dummy source and setup parameters established during the initial simulation is taken. The balloon diameter is measured and compared with the reference film. The SPS dummy source position is verified against the balloon's geometric center. Using the scale marked on the MammoSite catheter, the position of the catheter with respect to the patient's skin is recorded and compared with the initial setting. The treatment planning dwell time calculation is independently validated by using an AAPM TG 43^(14)^ one‐dimensional point source approximation in an Excel (Microsoft Corporation, Redmond, WA) spreadsheet to calculate the dose to the prescription point:

(2) $$D(r)=S_{K}\cdot \Lambda \cdot {\left(\frac{r_{0}}{r}\right)}^{2}\cdot g_{p}(r)\cdot \varphi_{an}(r)\cdot t,$$

where *D*(*r*) is the dose at the prescription distance $r;S_{k}$ is the air kerma strength of the source at reference distance $r_{0};\Lambda$ is the dose rate constant; $g_{p}(r)$ is the radial dose function; $\varphi_{\text{an}}(r)$ is the anisotropy function; and *t* is the planned dwell time.

## III. RESULTS AND DISCUSSION

In general, correlation of the balloon diameter between CT, planar appositional film, and surgeon's fill volume has been good (within 1 mm) and has held constant during treatment. For patient 2, the maximum diameter measured on the CT scan was 4.8 cm, which coincided with the balloon dimension on film. The mean diameter, 4.6 cm, corresponded with the surgeon's fill volume (within 1 mm). The fill volume provided by the surgeon for patient 4 was reported incorrectly.

The source dwell position defined by the SPS coincided with the initially defined dwell position and with the center of the balloon to within 1 mm. The independent dwell time validation obtained by Excel spreadsheet was within 3% of the BPS calculation. The position of the catheter with respect to the patient's skin held constant throughout treatment.

At the time of the CT scan, 30.4% of our implants either did not initially meet the RTOG 0413 acceptance criteria (21.7%) or showed a parameter that could be improved by manipulation (8.7%; Table 2). Intervention corrected or improved all but one of these discrepancies. Patient 23 was not planned because the minimum balloon surface–to–skin distance was 3 – 4 mm, and the implant was not able to be salvaged.

**Table 2:** Symmetry, conformance, and target volume coverage change with balloon manipulation^a^

Patient	Manipulation type	Symmetry change	Conformance change	Target volume coverage change
1	Patient sent back to surgeon to evacuate from the cavity a large hematoma that surrounded the balloon and to re‐implant the balloon	N/A	**Improved to 3.9% from** >90%	N/A
2a	Added 15 cm^3^ fluid to the balloon	Asymmetry increased to 3 mm from 1 mm	Increased to 5.6% from 5.2%	**Improved to 92.1% from 90.0%**
2b	Removed 5 cm^3^ fluid from the balloon, resulting in a net change of $+10\;{\text{cm}}^{3}$ to the original balloon volume	**Asymmetry decreased to 1.25 mm from 3 mm**	Improved to 4.2% from 5.6%	Improved to 93.8% from 92.1%
3	Added 10 cm^3^ fluid to the balloon	Unchanged at 1.5 mm	Improved to 1.5% from 2.3%	Decreased to 94.1% from 94.4%
4	Removed 10 cm^3^ fluid from the balloon	Asymmetry decreased to 1 mm from 2 mm	Increased to 4.9% from 4.4%	**Improved to 92.2% from 89.5%**
5	Added 10 cm^3^ fluid to the balloon and massaged the breast to remove air pocket at wound site	Asymmetry increased to 1 mm from 0.5 mm	Improved to 0.0% from 3.8%	Improved to 98.1% from 94.5%
6	Waited 2 days; most of the air resolved. Added 10 cm^3^ fluid to the balloon to reduce the remaining air	Unchanged at 0.5 mm	Improved to 1.9% from 8.4%	**Improved to 92.1% from 84.7%**

a Items in bold did not initially meet RTOG 0413 criteria.

**Figure 3:**
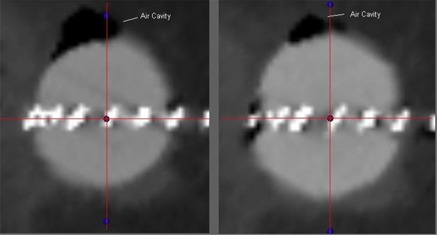
Pre‐ and post‐manipulation images of patient 2. The images are multi‐planar reconstructions of the computed tomography scan of the implant. The image on the left is the implant in its postoperative state. The image on the right is the implant after a net addition of 10 cm^3^ to the balloon volume. The size of the air pocket has been reduced, with an acceptable 0.25 mm increase in asymmetry.

**Figure 4:**
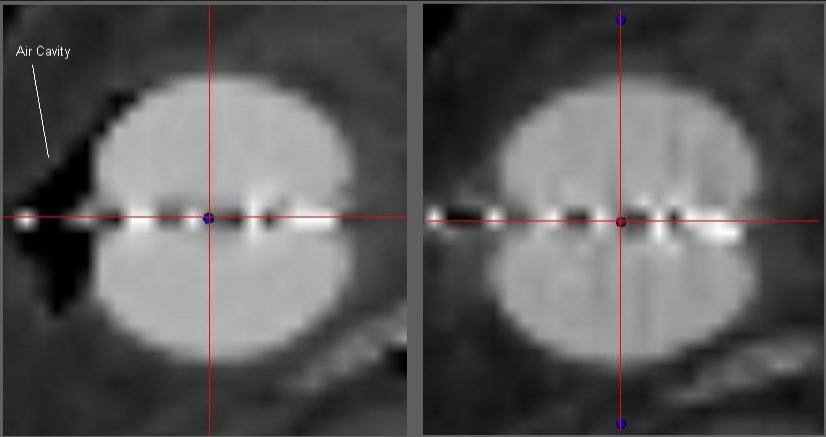
Pre‐ and post‐manipulation images of patient 5. The images are multi‐planar reconstructions of the computed tomography scan of the implant. The image on the left is the implant in its postoperative state. The image on the right is the implant after addition of 10 cm^3^ fluid to the balloon and massage of the implant area. The air pocket has been eliminated, with an acceptable 0.5 mm increase in asymmetry.

Figs. 3 and 4 show the pre‐ and post‐manipulation images for patients 2 and 5 respectively. The images are multiplanar reconstructions of the CT data set, illustrating the impact of two manipulation techniques: adding fluid to the balloon and massaging the implant site.

Table 3 lists the final RTOG 0413 geometric and dosimetric parameters for all patients in our series. Values are well within the protocol's guidelines for treatment.

**Table 3:** Radiation Therapy Oncology Group (RTOG) 0413 protocol parameters

Patient	Balloon symmetry (mm)	PTV_EVAL volume (cm^3^)	Tissue–balloon conformance (%)	*V*90 target volume coverage (%)	Balloon surface–to–skin distance (mm)	Maximum skin dose (%)	Volume lumpectomy / volume of ipsilateral breast (%)	Normal breast tissue dose–volume (cm^3^)	
								*V*150	*V*200
1	0.5	119	3.9	95.3	27	50	8.9	29.8	0.9
2	1.25	87.0	4.2	93.8	13	85	3.6	29.7	5.7
3	1.5	97.0	1.5	94.1	12	85	4.4	23.8	2.2
4	1.0	92.0	4.9	92.2	25	58	3.4	28.3	6.2
5	1.0	107	0.0	98.1	19	70	4.9	30.3	2.9
6	0.5	79.3	1.9	92.1	8	140	9.7	24.3	4.1
7	0.5	96.1	0.8	98.1	11	90	4.6	26.3	2.2
8	1.0	117	0.03	94.0	12	85	7.2	27.4	0.9
9	1.0	95.0	6.8	90.0	23	50	4.1	27.3	3.7
10	1.5	83.4	0.2	93.2	11	85	5.8	22.7	4.2
11	0.5	78.8	0.03	94.5	12	90	4.7	23.3	4.7
12	1.0	93.0	0.0	97.1	26	45	3.4	26.9	4.4
13	1.2	115	0.1	95.6	33	41	4.1	29.5	2.9
14	2.0	70.7	0.0	92.6	11	95	7.7	22.1	5.5
15	0.5	94.6	0.0	90.7	20	60	3.8	20.1	2.4
16	1.0	89.6	0.1	95.7	32	30	2.9	25.6	4.0
17	1.0	77.2	0.0	97.2	8	135	2.4	29.1	8.1
18	1.5	100.0	0.7	93.5	29	48	10.6	22.8	3.7
19	0.5	82.1	1.1	92.4	17	55	4.8	20.9	2.94
20	0.5	108	0.8	94.6	26	45	3.8	27.3	2.9
21	0.6	111	3.2	95.8	12.5	85	5.4	32.6	2.1
22	2.0	87	2.9	95.1	22	57	6.1	27.5	4.0
Average	1.0	94.5	1.5	94.4	19	72	5.3	26.3	3.7
Median	1.0	93.8	0.75	94.3	18	65	4.7	27.1	3.7
Standard deviation	0.48	13.70	1.96	2.21	8.01	28.62	2.23	3.36	1.72

Notably, although patients 2, 4, and 6 did not initially meet the RTOG dosimetric criteria for target volume coverage, they did meet the manufacturer's guidelines for treatment appropriateness. Performing full 3D CT‐based treatment planning with DVH analysis revealed the inadequate target volume coverage and the need for corrective action. Even with perfect conformance (no air or seroma), if the prescription point is placed 1 cm from the balloon surface, the target volume coverage will always be less than 100% because of source anisotropy. Asymmetry and poor conformance values reduce the target volume coverage, so that an implant with moderate conformance and asymmetry can be within the manufacturer's guidelines, but not meet the RTOG criteria.

## IV. CONCLUSIONS

Manipulating the cavity and adjusting the balloon volume may salvage an implant and assist in meeting the strict geometric and dosimetric criteria imposed by the RTOG 0413 protocol. In general, adding fluid increases tissue–balloon conformance and reduces symmetry. The surgeon's recorded fill volume should be verified. The RTOG 0413 criteria for MammoSite treatment planning are valuable for evaluating the quality of an implant, and undertaking the more extensive RTOG dosimetric and geometric analysis for non‐protocol patients may be appropriate.

## Supporting information

Supplementary Material Files
