# Supplementary material for: Clinical and dosimetric experience with MammoSite‐based brachytherapy under the RTOG 0413 protocol
Source: J Appl Clin Med Phys. 2007 Oct 24;8(4):176–84. doi: 10.1120/jacmp.v8i4.2654 (PMC5722622; doi:10.1120/jacmp.v8i4.2654)
Supplement: Supplementary file 1 — Supplementary Material Files [file ACM2-8-176-s001.doc]

Clinical and dosimetrical experience with MammoSite based brachytherapy under RTOG-0413 protocol

Abstract: MammoSite balloon brachytherapy is a relatively new technique for partial breast irradiation. This paper focuses on the treatment planning, dosimetry and quality assurance aspects of treatment based on the Radiation Therapy Oncology Group (RTOG) 0413 randomized prospective trial protocol. The usefulness of evaluating implants according to the full set of RTOG criteria versus the manufacturer’s guidelines for treatment appropriateness is investigated. We describe our methods to improve MammoSite balloon implants that would otherwise not comply with the protocol. The initially acquired CT images are evaluated for tissue conformance, balloon surface to skin distance and balloon symmetry. If the implant fails to meet the above criteria, corrective action such as delaying the CT scan, balloon manipulation, fluid volume adjustment is taken and the patient is re-scanned. If the corrective action appears to be successful, 3D treatment planning and DVH analysis is performed in order to evaluate the geometric and dosimetric parameters per the RTOG-0413 protocol. The parameters include: the volume ratio of the lumpectomy cavity to the ipsilateral breast, target volume coverage, tissue-balloon conformance, balloon symmetry, minimal balloon surface-skin distance, maximum skin dose, and normal breast tissue dose volume parameters: V150 and V200. 21.7% of our implants did not initially meet the RTOG-0413 acceptance criteria. Asymmetry and poor conformance values decrease the target volume coverage, so an implant with moderate conformance and asymmetry can be within the manufacturer’s guidelines but not meet the RTOG criteria. Through our intervention all but one were corrected. Manipulating the cavity and adjusting the balloon volume may salvage an implant and meet the strict geometric and dosimetric criteria imposed by the RTOG-0413 protocol.

**Keywords**— MammoSite®, Brachytherapy, Breast cancer, High Dose Rate, Partial Breast Irradiation

PACS number: 87.53.Jw
